# Opium Poisoning, Treated by Electricity and Belladonna

**Published:** 1877-01-20

**Authors:** Martin Burke

**Affiliations:** New York City


### OPIUM POISONING, TREATED BY ELECTRICITY AND BELLADONNA.

BY MARTIN BURKE, M D., OF NEW YORK CITY.

I would, with your permission, report a very interesting case of opium poisoning cured by electricity and belladonna.

Late one evening, when returning home from a distant visit, I was hurriedly addressed by a man who desired me to go immediately to see Mr. S., who had taken a sufficient quantity of opium to destroy life. While I was going for my battery, he called upon my friend Dr. Hanks, who resides beside me. We both hurried to the residence of our patient, and found him in a state of coma; pupils contracted to the size of a pin's head, and breathing somewhat stertorous. His respirations were four a minute ; his pulse was slow. His wife stated that he had taken the opium about forty-five minutes before. Dr. Hanks had meanwhile sent for a prescription of—

℞. Zinci sulphatis, gr. xxx
Fluidi extracti ipecacuanhæ, dr. iss which, having been procured, was immediately administered. It vomited our patient in about three minutes. A Dr. White had meanwhile arrived, and he proposed the administration of belladonna; accordingly, about the twenty-ourth of a grain of atropia was given hypodermically. Another mixture of zinc and ipecac was administered. It promptly vomited the patient, and, indeed, in the matter ejected we detected the smell of opium. A small quantity of food was also discovered.

Our patient was now partially conscious, and although we had introduced the tube of a stomach pump into his pharynx, he caught it between his teeth, and fearing that he would cut it through, we removed it. Our patient said he wished to die. His clothes were dragged upon his reluctant limbs, and at half-past eleven o'clock we commenced to walk our would-be suicide.

I now examined the bottle in which he had procured the preparation of opium. It was a two-ounce bottle, in which remained about a drachm of dark-looking fluid. I afterward learned from the druggist, to whom Mr. S. was known, that he had procured a two-ounce mixture containing equal parts of tincture opii and alcohol; of this only a drachm remained. We now administered large quantities of strong black coffee, part of which was vomited. At about quarter after twelve A.M., another one-twenty-fourth grain of atropia was administered, subcutaneously. Our patient was now conscious, but was unwilling to walk. Pupil's had dilated to almost normal size. More coffee was given. Half-past one o'clock, patient staggered and fell. We now proposed electricity, and accordingly I applied both poles of the battery over the situation of the phrenic nerves; it caused him to take a prolonged and deep inspiration. His respirations, with and without electricity, were counted ; with electricity, they were about nine per minute; without it, seven.

In order to keep him awake, or to awaken him, we occasionally applied the poles of the battery to the posterior cervical nerves. This aroused him at once ; he raised himself, and endeavored to escape from this sharp sting. We removed the poles, and again he sank into a state of coma. At about three o'clock atropia, grain one-twenty-fourth, was again administered. His pupils now became normal. We had previous to this never permitted our patient to sleep, using only electricity; but at four o'clock we permitted him to slumber, employing a somewhat weaker power to the phrenic nerves, thereby increasing the depth of the respirations, and, indeed, their frequency, in about the proportion of one to three a minute. In lengthening the respiratory force its effect was wonderful. Dr. Hanks and I relieved each other, remaining with our patient until about six o'clock. We now could easily arouse him, and accordingly it was deemed advisable to remove the battery. I should state, however, that the patient's respiration never, during our treatment, went below four, and never, even under the influence of electricity, went above ten.

Mr. S., being of strong physique, recovered rapidly. Three days after he attended to his business.—*Medical and Surgical Reporter.*

